# Exploring hydrodynamic cavitation for citrus waste valorisation in Malta: from beverage enhancement to potato sprouting suppression and water remediation

**DOI:** 10.3389/fchem.2024.1411727

**Published:** 2024-05-27

**Authors:** Georgios Psakis, Frederick Lia, Vasilis P. Valdramidis, Ruben Gatt

**Affiliations:** ^1^ Institute of Applied Sciences (IAS), The Malta College of Arts, Science and Technology (MCAST), Paola, Malta; ^2^ Metamaterials Unit, Faculty of Science, University of Malta (UM), Msida, Malta; ^3^ Laboratory of Food Chemistry, Department of Chemistry, National and Kapodistrian University of Athens (NKUA), Athens, Greece; ^4^ Centre for Molecular Medicine and Biobanking, University of Malta (UM), Msida, Malta

**Keywords:** hydrodynamic cavitation, citrus waste, green solvents, potato sprouting, water remediation, nitrates, nitrites, heavy metals

## Abstract

**Introduction:** The endorsement of circular economy, zero-waste, and sustainable development by the EU and UN has promoted non-thermal technologies in agro-food and health industries. While northern European countries rapidly integrate these technologies, their implementation in Mediterranean food-supply chains remains uncertain.

**Aims:** We evaluated the usefulness of hydrodynamic cavitation (HC) for valorizing orange peel waste in the fresh orange juice supply chain of the Maltese Islands.

**Method:** We assessed: a) the effectiveness of HC in extracting bioactive compounds from orange peels (Citrus sinensis) in water (35°C) and 70% (v/v) ethanol (−10°C) over time, compared to conventional maceration, and b) the potato sprouting-suppression and biosorbent potential of the processed peel for copper, nitrate, and nitrite binding.

**Results:** Prolonged HC-assisted extractions in water (high cavitation numbers), damaged and/or oxidized bioactive compounds, with flavonoids and ascorbic acid being more sensitive, whereas cold ethanolic extractions preserved the compounds involved in radical scavenging. HC-processing adequately modified the peel, enabling its use as a potato suppressant and biosorbent for copper, nitrate, and nitrite.

**Conclusion:** Coupling HC-assisted bioactive compound extractions with using leftover peel for potato-sprouting prevention and as biosorbent for water pollutant removal offers a straightforward approach to promoting circular economic practices and sustainable agriculture in Malta.

## 1 Introduction

Global citrus production surpasses 100 million tons annually, but approximately 50% of the fruit is deemed inedible, resulting in 60 million tons of waste per year (Mahato et al., 2019). EU and UN policies over the past decade have prioritized hierarchical food waste management, redistribution, and valorization to promote sustainable consumption and production, thus fostering circular economies (Teigiserova et al., 2020; Carlsen and Bruggemann, 2022). Emerging green technologies, such as hydrodynamic cavitation (HC), offer promising solutions for water softening (Anaokar and Khambete, 2021), chemical/dye inactivation (Zupanc et al., 2013; Bandala and Rodriguez-Narvaez, 2019) and non-thermal food processing (Sun et al., 2022).

In HC, device contractions induce turbulence, elevating kinetic energy at the cost of hydrostatic pressure until it matches (or falls below) the liquid’s vapor pressure (Carpenter et al., 2017). Flashing liquid forms cavitation bubbles, expanding upon pressure recovery. Upon collapse, bubbles emit energy waves, microjets, and reactive oxygen species, inducing chemical and physical changes in the treated material (Meneguzzo and Zabini, 2021). In citrus waste, such changes manifest as tissue loosening (Singhal and Hulle, 2022), improved dispersibility and functionality of pectin-biopolymers and antioxidants (Meneguzzo et al., 2019; Chu et al., 2022), as well as pectin methyl esterase inactivation (Arya et al., 2021) and natural colourant release (Ciriminna et al., 2020a). Most importantly, HC-induced changes are achieved over short yet intense treatments, economizing on energy, and preserving the stability/functionality of heat-sensitive core food components (Arya et al., 2023).

Typically, citrus waste valorization involves several steps, i.e., drying and pulverizing the peel to reduce water activity and increase surface area, mixing the dried peel with green solvents like water or ethanol, technology-assisted extraction, content determination, chemical identification, and purification of main compounds, and finally processing and valorization (Supplementary Figure S1) (Panda and Manickam, 2019; Nanda et al., 2021; Hessel et al., 2022). Extracted bioactive compounds, including flavonoids, terpenes, phenolics, essential oils, and carotenoids, enhance beverages, dairy, and baked goods, providing nutritional value and shelf-life extension due to their antimicrobial properties (Mohsin et al., 2022; Saini et al., 2022). Pectin acts as a thickener, stabilizer, and offers antimicrobial benefits (Meneguzzo et al., 2019; Ciriminna et al., 2020b), while extracted sugars and carbohydrates act as prebiotics, promoting beneficial microorganism growth (Gómez et al., 2014; Mazzucotelli and Goñi, 2023). Citrus peel can also undergo biorefinery processes to yield various products like acids, bioethanol, biomethane, xanthan, and curdlan gum (Mohsin et al., 2022). Additionally, it can be converted into activated biochar for ammonium ion removal (Tan et al., 2023), activated hydrochar for CO_2_ uptake (Deepak et al., 2023), or used as an alternative to nitrate/nitrite preservatives in fermented meats (Calderón-Oliver and López-Hernández, 2022).

In 2013-2014, the Maltese Islands produced 1,800 tonnes of citrus, yet over 80% of the island’s needs are fulfilled through imports (Parliamentary secretary for agriculture and fisheries and animal rights Malta, 2018). Locally grown oranges are typically consumed fresh or in juice form. Collected orange peel waste is used as livestock feed (Lalramhlimi et al., 2022) but albeit nutritious, it poses risks of acidosis in livestock due to its acidity (Alnaimy et al., 2017). Moreover, direct use of orange peel as fertilizer (Attard et al., 2019) or improper composting practices may lead to soil acidification and disrupt bacterial populations. Excessive use of manure can exacerbate the imbalance in the nitrogen cycle, increasing soil nitrates (Abascal et al., 2022) and adversely affecting groundwater quality. Overall, agricultural malpractices and intensive land use have significantly compromised soil and groundwater quality in Malta (Hartfiel et al., 2020).

Here we examine HC as a method for extracting bioactive compounds from orange waste peel in Malta, focusing on its effectiveness within the fresh juicing industry. We discuss the scientific data supporting HC extraction’s ability to recover valuable compounds from citrus waste and propose a citrus-waste valorization scheme that encourages sustainable practices, aligning with broader national economic and environmental goals.

## 2 Materials and methods

### 2.1 Drying procedure

We evaluated the performance of three drying techniques—microwave (MW; Midea, United States), hot-air (FOD; Zilan, France), and freeze-drying (FRD (N-series); Scientz, China)—for citrus peel obtained from freshly squeezed oranges (*Citrus sinensis*) sourced from Mgarr Farms and Koperattivi Malta. Three different lots of orange peel were sourced: Valenciana (DMA farms, Egypt), Valenciana (El Gebaly Fruit Company, Egypt) and Navel (F.C.C.S Limited, Malta). Samples from each lot were subjected to the following process: peel pieces (albedo, flavedo, and segment walls) were cut to 1–3 mm and stored at −80°C before drying. MW drying involved 175 W [ouput determined as in (Buesa, 2002)] microwave heating for 5 min followed by 10 min cooling at room temperature to prevent Maillard reactions (Ahmad and Langrish, 2012). FOD drying was carried out at 35°C, while FRD was conducted under vacuum (110 kPa) until moisture content and water activity were reduced to <0.2. The dried peel was pulverized (10 s bursts, 30 s cooling), sieved through 100 μm and 50 μm pore-size membranes for uniform particle size distribution. Particles failing to pass through the smaller sieve were sealed and stored for future use. For determining the influence of the drying process in the exhibited antioxidant activities (Section 2.4) each lot provided a replica. For subsequent works, the remaining peel from the three lots was dried using the most effective drying method and the powders were combined to generate a single homogeneous stock. Thus, subsequent extractions and analytical tests were conducted in technical triplicates.

### 2.2 Green solvent extractions

#### 2.2.1 Solvent-mediated extractions

70% (v/v) ethanol (Biochem Chemopharma, France) was the selected green solvent (as was previously identified as an optimal green solvent for at least total phenolic extraction by maceration (Mahato et al., 2019; Viñas-Ospino et al., 2023)). Drying effectiveness was primarily based on the process’s ability to preserve the ethanol-extracted biocomponents and/or to improve their antioxidant activities, relative to fresh peel. Dried and/or fresh peel was mixed with 70% (v/v) ethanol, at a 1:40 solid-to-liquid ratio (g/mL) (Selahvarzi et al., 2022), with mild stirring (Stuart Scientific, UK; 100 rpm) at 35°C for 2 hours. Samples were taken at 15, 30, 60, and 120 min of extraction time for determination of their antioxidant activities (Section 2.4). All extractions and analytical experiments, including the generation of standard calibration curves were conducted in technical triplicates, unless otherwise stated.

#### 2.2.2 HC-mediated extractions

For HC-mediated extractions, we opted for water (the greenest of solvents) and 70% (v/v) ethanol (Mahato et al., 2019; Viñas-Ospino et al., 2023). Dried peel was enclosed in a 50 μm muslin bag and inserted into a 200 mL double-walled Scott bottle fitted with a two-port screw cap (Supplementary Figure S2A). Water or 70% (v/v) ethanol was added to the bottle at a 1:40 solid to liquid ratio. The screw-cap ports created a closed system (minimizing loss of volatile D-limonene) for continuous feeding into the HC-device. The double-jacketed container circulated coolant, maintained at the desired temperature by a chiller unit (WCR-P12; Witeg, Germany). Water extractions were at 35°C (to minimize thermal effects on the stability of antioxidants), and for ethanol, the temperature was set at −10°C to prevent flashing. Inlet and outlet pressures were monitored with digital sensors, and samples were collected at 15, 30, 60, and 120-min intervals. The choice of the time-range was simply based on the industry requirement for short processing times and energy consumption efficiency.

HC extractions were performed using a counter-rotational system (WHARPS Technologies, Malta), featuring two co-axially positioned rotors within a truncated conical chamber (Supplementary Figures S2B, S2C; Isopo, 2010). A maximum rotational frequency of 0.168 MHz facilitated bubble implosion at communication zones between rotor orifices and slots. The effectiveness of HC extractions was evaluated with both rotors operating at 50%, 75%, and 100% of their maximum frequency (the higher the frequency the more intense the cavitation). A small peristaltic pump modulated the liquid flowrate, achieving intercept cavitation numbers of 0.33 (50%), 0.29 (75%), and 0.25 (100%) for water at room temperature and 0.58 (50%), 0.55 (75%), and 0.50 (100%) for 70% (v/v) ethanol at −10°C. Cavitation numbers were derived as in (Omelyanyuk et al., 2022).

### 2.3 Determination of total reducing sugar, phenolic, and flavonoid contents

The total reducing sugar content of extracts was determined spectrophotometrically (UV-2600, Shimadzu, Japan) at 540 nm using the 3,5-dinitrosalicylic acid (DNS; ThermoFischer Scientific, United States) method, following (Khatri and Chhetri, 2020), with no modifications. Calibration curves of D-glucose (Biochem Chemopharma, France; 4.75–600 μg/mL) in Milli-Q water and 70% (v/v) ethanol were used for quantification, yielding R^2^ values of 0.997 and 0.996, respectively. Sugar contents were expressed as mg D-glucose equivalents (DGE)/g dry matter. Polyphenolic content was determined spectrophotometrically at 765 nm using the Folin-Ciocalteau (MP Biomedicals, France) method as per (Waterhouse, 2003), with no modifications. Total phenolic content was quantified using calibration curves of gallic acid (Apollo Scientific, UK; 10–250 mg/L) in Milli-Q water and 70% (v/v) ethanol, resulting in R^2^ values of 0.999, and expressed as mg gallic acid equivalents (GAE)/g dry matter. For total flavonoid contents, the method of (Mahboubi et al., 2013) was followed without modifications. Calibration curves of quercetin (VWR, France; 0.05–1.5 mg/mL) in Milli-Q water and 70% (v/v) ethanol were used for quantification, with R^2^ values of 0.999, and results were expressed as mg quercetin equivalents (QE)/g dry peel.

### 2.4 Determination of extract antioxidant activities

Antioxidant activities were determined using the: a) 2,2-diphenyl-1-picrylhydrazy (DPPH; Cayman Chemical, USA) radical, b) 2,2'-azino-bis(3-ethylbenzothiazoline-6-sulfonic acid (ABTS; Rockland, USA) radical cation (ABTS^•+^), and c) the H_2_O_2_ (Biochem Chemopharma, France) scavenging assays with no modification from (Shehata et al., 2021; Al-Amiery et al., 2015), for DPPH/ABTS^•+^ and H_2_O_2_ assays, respectively. For DPPH and ABTS^•+^, activities were expressed as mg Trolox (Cayman Chemical, USA) equivalents (TE)/g dry matter (prepared in 70% (v/v) ethanol), whereas for H_2_O_2_ scavenging, activities were expressed as mg ascorbic acid (Biochem Chemopharma, France; prepared in Milli-Q water) equivalents (AAE)/g dry matter. Standard Curves over the 0–60 μg/mL for Trolox and 0–400 μg/mL for ascorbic acid were constructed in Milli-Q water (with R^2^ values of 0.995, 0.995, 0.994, for DDPH, ABTS^•+^, and H_2_O_2_, respectively), and/or in 70% (v/v) ethanol (with R^2^ values of 0.995, 0.997, 0.999, for DDPH, ABTS^•+^, and H_2_O_2_, respectively).

### 2.5 Investigation of inhibition of spring potato sprouting

Following completion of the conventional extraction in 70% (v/v) ethanol and the HC-mediated extractions in water, the remaining peel was collected, and aseptically dried. The powdered peel was then applied with a sterile cloth on freshly harvested, washed, and dried spring potatoes. Peel-treated and untreated potatoes were placed in an aluminium foil box, covered with 230 gsm paper lid, and incubated at 35°C and 30% relative humidity for 30 days. Eye formation, and sprouting was followed over that period in triplicates as previously described (Thoma et al., 2022).

### 2.6 Fixed bed colum-scale removal of Cu^2+^, NO_2_^−^, and NO_3_^−^

0.9 g of unprocessed or HC-processed powdered peel was mixed with 1 mL of CuSO_4_ (MP Biomedicals, France; 5–80 mg/L; pH 5.5) or NaNO_2_ (Biochem Chemopharma, France; 1–500 mg/L; pH 7.0) or NaNO_3_ (Biochem Chemopharma, France; 3–100 mg/L; pH 6.0) solutions for 60 min at room temperature with mild shaking. The suspensions were then transferred to polypropylene tubes with a 50 μm-diameter cloth filter at the bottom to prevent adsorbent loss and clogging. Flowthroughs were collected and analyzed spectrophotometrically. CuSO_4_ absorbance was monitored at 635 nm, while NO_2_^−^ concentrations were determined using the Griess-Ilosvay assay. Nitrite absorptions were subtracted from total nitrate and nitrite absorptions, monitored at 220 nm, to obtain NO_3_^−^ concentration (Carvalho et al., 1998).

### 2.7 Curve fitting and statistical analyses

Histograms were generated using GraphPad Prism version 10 for windows (GraphPad Software, United States). Differences were statistically evaluated using the two-way ANOVA multiple comparisons option of the software. The Langmuir (Ebrahimi-Gatkash et al., 2017), and the natural logarithmic transformation of the Freundlich isotherm (Degefu and Dawit, 2013) were fitted to the obtained adsorption data using the same software.

## 3 Results and discussion

### 3.1 Hot air-drying can be as efficient as freeze-drying

Selection of a universal drying technique is challenging since its effectiveness is product, food-matrix, and food-layer dependent. Sun-drying preserves bioactive compounds in orange peel but is time-consuming and risks flavonoid oxidation (Li et al., 2006). MW and infrared heating offer faster drying rates than conventional methods, especially when combined with vacuum drying at 50°C (Bozkir et al., 2021). Tray or hot-air drying are popular due to simplicity and low costs but compromise bioactive compound content at temperatures ≥50°C, necessitating vacuum conditions for increased drying rates. Lyophilization achieves high retention of bioactive compounds and preserves natural colors and aromas, but its high costs limit its adoption by small- and medium-sized businesses (SMEs) (Li et al., 2006; Bozkir et al., 2021).

In harmony with previous works, drying did not only achieve preservation of the fruit through reduction of its water activity but also allowed for extract-enhanced antioxidant activities relative to the fresh peel (Supplementary Figure S3) (Özcan et al., 2021). Though all our tested drying methods resulted in similar extract antioxidant activities, as determined by the DPPH (Supplementary Figure S3A) and ABTS^•+^ (Supplementary Figure S3B) assays, FOD and FRD exhibited at least 2.2-fold higher H_2_O_2_ scavenging activities than MW-dried extracts (Supplementary Figure S3C). Microwave heating at 175W, albeit being the least energy consuming (0.35 kWh over 2 h), encouraged heat transfer processes from inside out, affecting the peel’s vitamin C content as well as its potential antioxidant properties by at least 2-fold, as previously reported (Alibas and Yilmaz, 2022). Of the drying processes FRD has been consistently reported to achieve the highest retention of antioxidant activities in fruits, with convective and/or conductive heating processes at 50–100°C achieving lower retention due to thermos-oxidative damage of the available bio-compounds (Kittibunchakul et al., 2023). In this work, FRD’s energy consumption amounted to 25.61 kWh over 26 h, whilst a conventional food drier operating at 35°C consumed 2.66 kWh over 18.7 h. Maintenance of dry-heating at 35°C, appeared to be gentler to the more heat-sensitive vitamin C, as well as flavonoids (Supplementary Figure S3C), making FOD equally effective to FRD and providing a viable economic option to SMEs.

### 3.2 Conventional vs. HC-assisted extractions; thoughts on functionalisation

**FIGURE 1:**
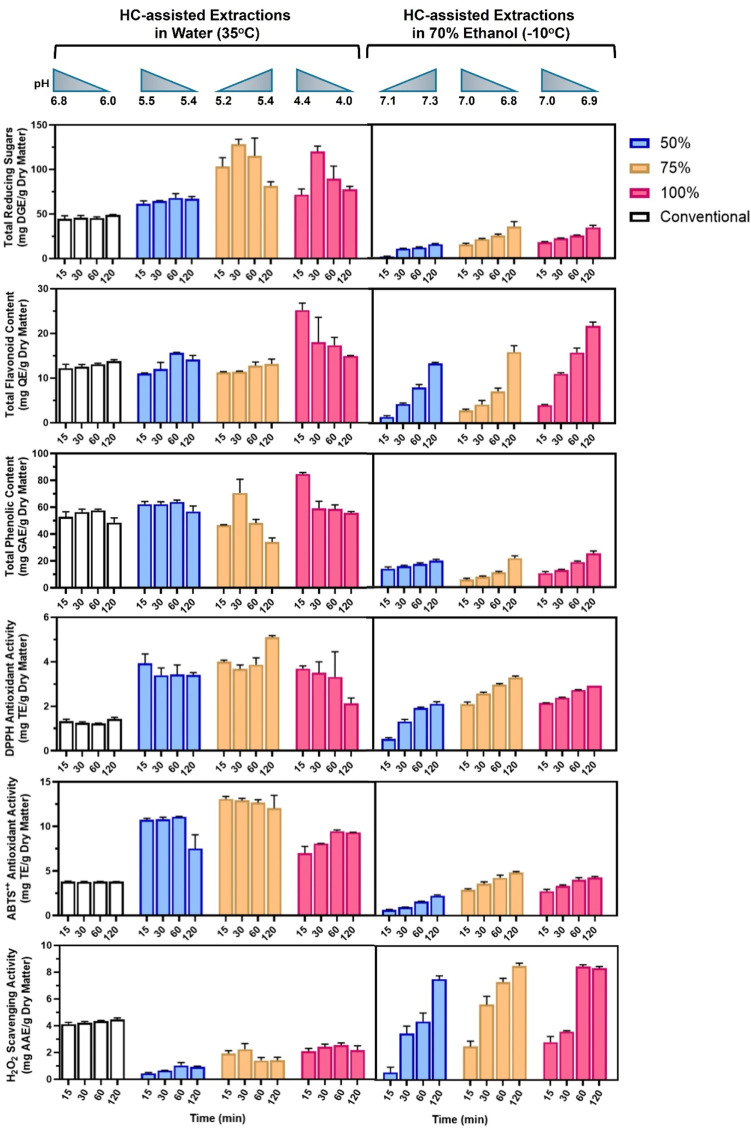
Orange peel waste extract contents and antioxidant activities. Top-to-Bottom: total reducing sugars, total flavonoids, total phenolics, DPPH, ABTS^•+^, and H_2_O_2_ scavenging. HC-assisted extractions were conducted in water (35°C, left column) and 70% (v/v) ethanol (−10°C, right column). White histograms; maceration in 70% (v/v) ethanol, blue histograms; HC at 50% rotational frequency, orange histograms; HC at 75% rotational frequency, and magenta histograms; HC at 100% rotational frequency. pH changes with time for the corresponding treatments are indicated. Error bars denote the STDEV of triplicate measurements.

Extractions in 70% (v/v) ethanol, with mild stirring at 35°C, revealed average TPC and TFC contents of 53.9 ± 4.1 mg GAE/g, and 12.9 ± 0.7 mg QE/g, respectively (Figure 1, conventional), in harmony with previous studies reporting contents of 34.6 ± 2.1 mg GAE/g TPC (1:25 solid-to-70% (v/v) ethanol, 37°C, 1 h; (Liew et al., 2018), and 10 mg QE/g TFC (1:10 solid-to-80% (w/v) ethanol, 35°C, 30 min; (Mhiri et al., 2016). At 35°C, the pH of 70% (/v) ethanol was close to neutrality (pH 6.82 ± 0.12), improving the extraction yield of phenolics and possibly polyphenols (Haya et al., 2019), relative to flavonoids whose extraction yields improve under more acidic conditions (for a review see (Chaves et al., 2020)) (Figure 1, left column). TPCs correlated with TFCs and antioxidant activity (Supplementary Figure S4A; Kassambara, 2017), with DPPH reflecting the -OH contributions of the phenolics, and the ABTS^•+^ accounting for the presence of flavanones, and pyrogallol structures [as also described in (Platzer et al., 2021)], reflecting the flavonoid as well as phenolic contributions possible better than DPPH (Floegel et al., 2011). Indeed, 70% (v/v) ethanol at 35°C achieved bioactive-compound extraction from peel with ABTS^•+^ activity of 3.83 ± 0.01 mg TE/g within the first 15 min, and a DPPH activity of 2.29 ± 0.03 mg TE/g (Figure 1, left column). Our observed ABTS^•+^ activity was at least 2-fold higher than that reported in 50% (v/v) ethanol, over 30 min (Ashraf et al., 2024). In contrast, the extracts’ DPPH activity was at least 8-fold lower than that reported by Liew *et al.* (Liew et al., 2018), but the authors conducted their extraction over 72 h, suggesting that maceration with mild heating needs to be prolonged for achieving higher extraction yields.

For the HC-mediated peel extractions in water, the higher the rotational speed (i.e., the lower the cavitation number and the more efficient the cavitation) the higher the TRS, TPC, and TFC contents, with longer exposures (>30 min) compromising extraction yields (particularly for phenolics and total reducing sugars) (Figure 1, left column). This is consistent with the breakdown of organics following extensive cavitation as previously described (Montusiewicz et al., 2017). The more extensive the cavitation, the lower the recorded pH (Supplementary Figure S4A, negative correlation). The more acidic the extracts, the higher the yields and their antioxidant activities (Supplementary Figure S4A, negative correlation), in harmony with previous observations (Hegazy and Ibrahium, 2012). Although DPPH and ABTS^•+^ antioxidant activities corelated positively, their relationship to the TPC and TFC contents appeared reverse (Supplementary Figure S4A). This is to highlight that: i) within the TPC and TFC contents, only a certain percentage of compounds possesses the structural characteristics for exhibiting antioxidant activity (Aaby et al., 2004; Czech et al., 2020), ii) following the HC-induced peel damage, there is the potential of H_2_O_2_ production (Buron-Moles et al., 2015), dependent on the Fe content of the peel (Czech et al., 2020), putatively responsible for the oxidation of those bioactive compounds (Wu et al., 2015), and iii) depending on the bio-compound structure, oxidation may enhance or quench their antioxidant activities (Speisky Cosoy et al., 2022), as evidenced by the H_2_O_2_ scavenging capacity of the HC-extracts in water, relative to the conventional extraction (Figure 1, left column).

In contrast, HC-peel treatments in 70% ethanol at −10°C eliminated heating and abrupt pH changes, enhancing extraction efficiency, as previously reported (Mhiri et al., 2015). At −10°C, cavitation bubble formation was weaker (higher cavitation numbers) with longer exposure times correlating positively with extraction yields (Supplementary Figure S4B). Consequently, by elimination of thermal effects, weaker cavitation was effective over prolonged treatments. Additionally, clear positive correlations emerged between the recorded antioxidant activities (Supplementary Figure S4B). Total flavonoids were the major contributor towards the DPPH, ABTS^•+^ and H_2_O_2_ scavenging activities under slightly acidic conditions (Figure 1, right column). However, TPC correlated negatively with DPPH, suggesting that cold temperatures do not facilitate release of phenolics with strong antioxidant activities (Supplementary Figure S4B). Neutral-to-alkaline conditions contributed positively to the measured DPPH activity (Supplementary Figure S4B), which was almost 3-fold higher than that of the conventionally treated extracts (Figure 1, right column). Alkaline oxidation of flavonoids like quercetin, has been already reported to generate metabolites whose antioxidant capacity surpassed that of their precursor (Speisky et al., 2023). Thus, prolonged HC-assisted extractions in ethanol under cold conditions (neutral-to-alkaline pH) preserve and/or enhance the bioactivity of flavonoids while keeping the sugar content of the extracts low. In future works, we will define the nature and amounts of the extracted compounds in the selected and/or additional solvents by UPLC-MS. Also, we will proceed by assessing the potential of ultrasound- and pulsed electric field (PEF)-assisted extractions, as combinations of these non-conventional treatments with HC are likely to enhance extraction yields with shorter processing times.

Evidently, different HC treatments produce varying antioxidant contents, influenced by the raw material, as well as the interdependency between pH, temperature, and oxidation. Thus, effective integration of HC in food processing requires a clear understanding of the desired product, since design considerations determine the type and quantity of antioxidants needed for value addition, shelf-life extension, or sensory enhancement. State (e.g., powder or liquid) and cooking method further impact antioxidant bioavailability. For instance, to fortify cookies, peel waste (subjected to brief HC-treatments) can enhance antioxidant release when added as dried powder to flour, optimizing bioavailability during baking. In contrast, dairy fermentations may benefit from controlled release of antioxidants into the food matrix, using encapsulation-based methods (Adinepour et al., 2022).

### 3.3 Potato sprouting prevention

The use of essential oils in prevention of potato-sprouting has been well described with products comprising D-limonene currently approved as sprouting suppressants (Thoma and Zheljazkov, 2022). HC-assisted extraction of D-limonene from waste orange peel has also been explored as a more sustainable approach to conventional extractions (Meneguzzo et al., 2020). However, little is known about the overall potential of dried and/or HC-processed peel as a potato-sprouting suppressant, considering that it comprises both free and bound compounds with antioxidant propensity (Oboh and Ademosun, 2012). Following HC-assisted extractions, we collected the processed peel, air-dried it and investigated its potential as a sprouting suppressant (Section 2.5). We found that the processed peel exhibits potato-sprouting suppressant abilities, and that the more intense the HC-treatment (higher extract antioxidant activities, and potential plant-tissue damage reflecting reduced bound antioxidants) the weaker the prevention (Supplementary Figure S5). Thus, powdered peel, previously subjected to moderate processing by HC, can be further valorised as a potato-sprouting suppressant, providing a cost-effective preservation method, contributing to sustainability that is an elemental aspect of process intensification.

### 3.4 Water remediation

**FIGURE 2:**
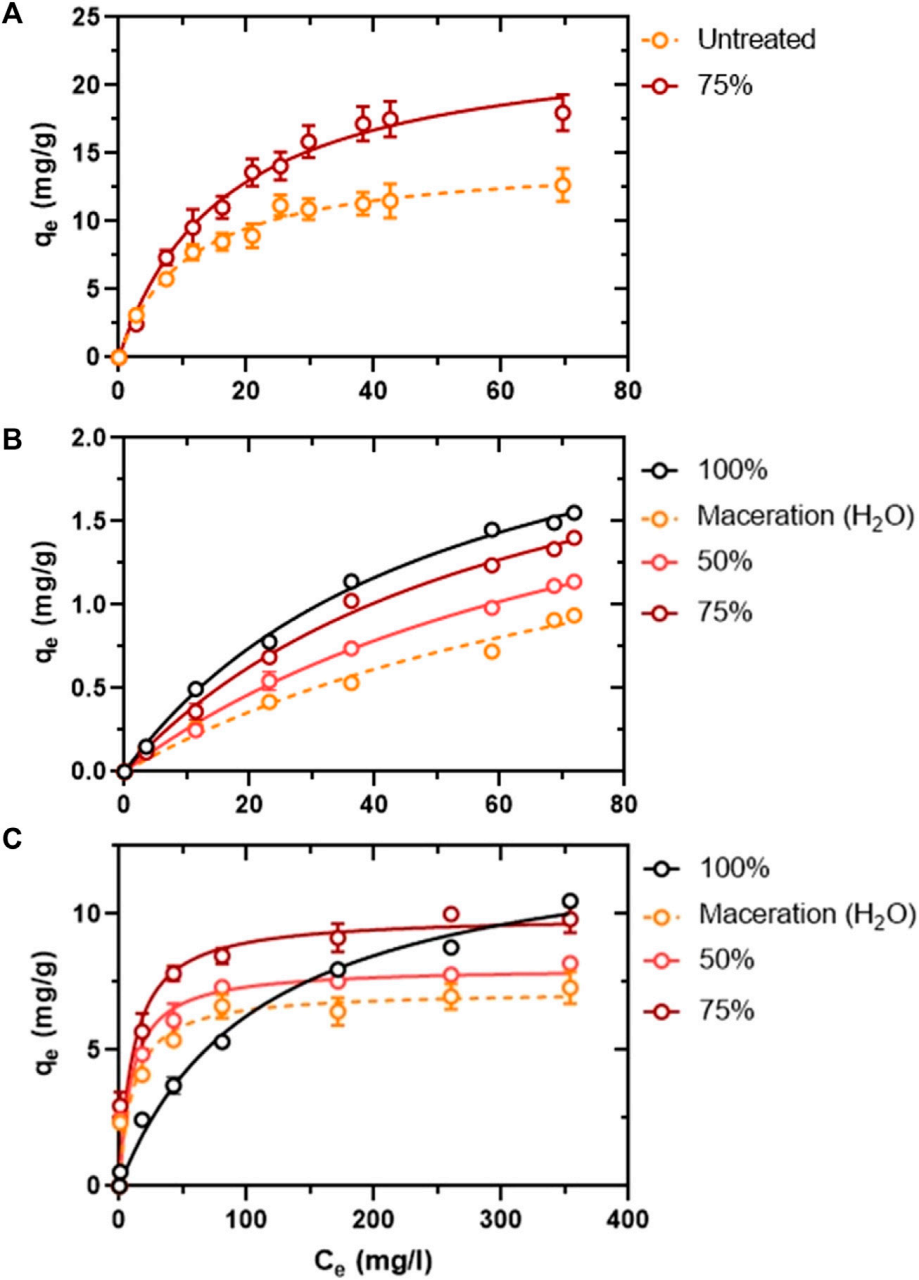
Non-liner Langmuir isotherm following adsorption of Cu^2+^ to untreated and HC-treated (water, at 75% rotational frequency, for 2 h) peel. q_e_ and c_e_, refer to the adsorption capacity of the peel and the concentration of ions used at equilibrium, respectively **(A)**. Non-liner Langmuir isotherm following adsorption of nitrates to water-macerated and HC-treated (water, at the specified rotational frequencies, for 2 h) peel **(B)**. Non-liner Langmuir isotherm following adsorption of nitrites to water-macerated and HC-treated (water, at the specified rotational frequencies, for 2 h) peel **(C)**. Error bars denote the STDEV of triplicate measurements.

In countries lacking established juice-plant processing, hydrodynamic cavitation (HC) aids in extracting essential oils and bioactive compounds from peel waste while offering preliminary physicochemical treatment for further valorization. Unprocessed peel is a low-to-moderately effective bio-sorbent, but its adsorption is enhanced following physicochemical, or biological treatments (Michael-Igolima et al., 2023). This enhanced adsorption makes it effective at removing contaminants such as heavy metals, nitrates, and ammonia from water (Dey et al., 2021; Mahato et al., 2021). We have assessed the adsorption of copper (Figure 2A), as well as nitrates (Figure 2B) and nitrites (Figure 2C) on unprocessed and HC-processed peel, with biosorption parameters reflecting favourable binding (Supplementary Tables S1, S2) in harmony with previous works (Feng et al., 2009; Izquierdo et al., 2013; Abdulrazak, 2016; Romero-Cano et al., 2016; Amin et al., 2017; Surovka and Pertile, 2017; Özkan et al., 2017; Amin et al., 2019; Safari et al., 2019; Kumar and Raju, 2020; Amirsadat et al., 2022; Shahaji et al., 2023). Though further HC-treatment and pH optimisations are required to maximise copper and nitrate binding, these preliminary works demonstrate that HC pre-treatments even under suboptimal pH can provide a level of physical peel modification essential for the adsorption of the tested chemicals. Interestingly, adsorption of nitrite (whose reactivity and toxicogenic propensity are stronger than nitrate) to the HC-processed peel appeared more favourable than nitrate (Supplementary Tables S1, S2; increased nitrite biosorption parameters relative to nitrate).Thus, utilisation of the adsorptive strength of orange peel in the filtration of the Maltese ground water, considering its deterioration (Hartfiel et al., 2020), can assist in the reduction of heavy metal, as well as nitrate, nitrite, and ammonium, to levels below those suggested by the EU directive (Psakis et al., 2023). With demonstrated reusability in nitrate/ammonium and iron-oxide removal, orange peel filtration offers a cost-effective solution for water remediation in Malta’s agricultural sector (Oteng-Peprah et al., 2018; Dey et al., 2021; Praipipat et al., 2023).

## 4 Leaping three mountains with one jump

Despite HC’s potential in process intensification (Katariya et al., 2020), its adoption by SMEs as a green technological processing step remains hesitant, despite offering lower capital costs compared to PEF (Avdieieva et al., 2023). HC, with various available devices (Sun et al., 2022), presents a cost-effective solution for valorizing orange peel waste. The cavitator used in this study allows easy compartment exchange (food grade) without additional pumping, ensuring compliance with food-processing legislation. Short HC treatments in water effectively extracted antioxidants, facilitating juice product functionalization. Concerns over CO_2_ footprint and capital costs associated with sophisticated freeze-drying equipment can be mitigated by solar-panel freeze-drying technologies (Zhou et al., 2021), potentially subsidized by government schemes. Alternatively, SMEs can opt for more economical hot-air/tray drying methods. Studies comparing the cost-benefit analysis of HC-produced foods to conventional processes are lacking. However, PEF-based analyses indicate higher production costs (Sampedro et al., 2013), potentially leading to higher product prices, which consumers may be unwilling to pay (Aschemann-Witzel and Zielke, 2017). Nevertheless, limited availability of niche functionalized sustainable foods may encourage consumer purchasing (Maojie, 2023). Dried orange peel waste can prevent potato sprouting for up to 3 weeks, paralleling beverage functionalization to peel drying processes. For Malta, extended potato shelf life is crucial for maintaining profitability in export markets like the Netherlands, Germany, Switzerland, and the UK (Parliamentary secretary for agriculture and fisheries and animal rights Malta, 2018), where spring potatoes are exported. Profits generated can help offset HC-functionalized beverage production costs, making these products more affordable for consumers. Moderately acidic soils (pH 5.0–6.5) are ideal for potato growth. Utilizing orange peel waste’s biosorbent capacity for pollutants like ammonium, nitrates, nitrites, and heavy metals in constructing biosorbent or biochar fixed-bed columns can improve irrigation water quality, enhancing soil conditions for potatoes. This proposed coupling of processes offers a straightforward approach to initiating circular economic practices in Malta, promoting sustainable agriculture.

## 5 Conclusion and final thoughts

In this work, we bench-scale evaluated the performance of a counter-rotational HC device in extracting bioactive compounds from unprocessed orange peel waste, using water and 70% (v/v) ethanol with heating <40°C and at −10°C, respectively. In agreement with previous works, this study highlights the potential of HC-mediated peel waste treatments for bio-compound extraction in green solvents for industrial processing. Furthermore, we have demonstrated that: i) hot-air drying can be a cost-effective alternative for orange peel waste drying, instead of the more costly freeze drying, ii) prolonged HC-assisted extractions in water, at high cavitation numbers, can mechanically damage and/or oxidise the bioactive compounds, with flavonoids and ascorbic acid appearing more sensitive to the treatments, iii) cold extractions in 70% (v/v) ethanol, preserve the nature of flavonoids and those organic acids that contribute to increased radical scavenging, and iv) HC-processing provides an adequate level of physical peel modification, facilitating its use as a potato suppressant and biosorbent for copper, nitrate and nitrite.

Regarding the selection of appropriate conditions for industrial processing, given the interdependency of cavitation effects, pH, temperature, and oxidative damage, we have further advised on the necessity of a clear understanding of the food-product design process, as the product will dictate the groups of antioxidants required for functionalisation, and as such the directions for optimisations. With studies suggesting consumer acceptability for cavitation-functionalised beverages (Katariya et al., 2020) main challenges for HC adoption include processing optimisations and lack of regulatory legislation (Priyadarshini et al., 2019). Policy making is hindered by insufficient research on microbiology and toxicology. Factors influencing microbiology and toxicology include material, solvent, reactor type, and processing conditions. Given recent systematic research, establishing a database detailing extraction conditions, bio-compound contents, HC-processing details, and toxicological and microbiological profiling (Ben-Othman et al., 2020) is crucial. Availability of such information can drive future extraction strategies, involving the use of hybrid technologies that enable the development of synergistic effects over shorter processing times. Such tools will aid policy making and incentivize sustainability practices (Teigiserova et al., 2020), encouraging SEMs to embrace innovative green technologies in food production.

## Data Availability

The original contributions presented in the study are included in the article/Supplementary Material, further inquiries can be directed to the corresponding authors.
