# Supplementary material for: Exploring hydrodynamic cavitation for citrus waste valorisation in Malta: from beverage enhancement to potato sprouting suppression and water remediation
Source: Front Chem. 2024 May 27;12:1411727. doi: 10.3389/fchem.2024.1411727 (PMC11163080; doi:10.3389/fchem.2024.1411727)
Supplement: Supplementary file 1 [file DataSheet1.docx]

Supplementary Material

Exploring Hydrodynamic Cavitation for Citrus Waste Valorisation in Malta: From Beverage Enhancement to Potato Sprouting Suppression and Water Remediation

Georgios Psakis^1,2*^, Frederick Lia^1,2^, Vasilis P. Valdramidis^3^, Ruben Gatt^2, 4*^

# ^*^Correspondence: Corresponding Authors: georgios.psakis@mcast.edu.mt; ruben.gatt@um.edu.mt

# List of Supplementary Tables

**Supplementary Figure S1**………………………………………………………………………….....2

**Supplementary Figure S2**………………………………………………………………………….....3

**Supplementary Figure S3**………………………………………………………………………….....4

**Supplementary Figure S4**……………………………………………………………………….…....5

**Supplementary Figure S5**……………………………………………………………………….…....6

**Supplementary Table S1**. Isotherm parameters for NO_3_^-^ & NO_2_^-^ adsorption onto orange peel waste ……..………………………………………………………………………………………….……….7

**Supplementary Table S2**. Isotherm parameters for Cu^2+^ adsorption onto orange peel waste……......8


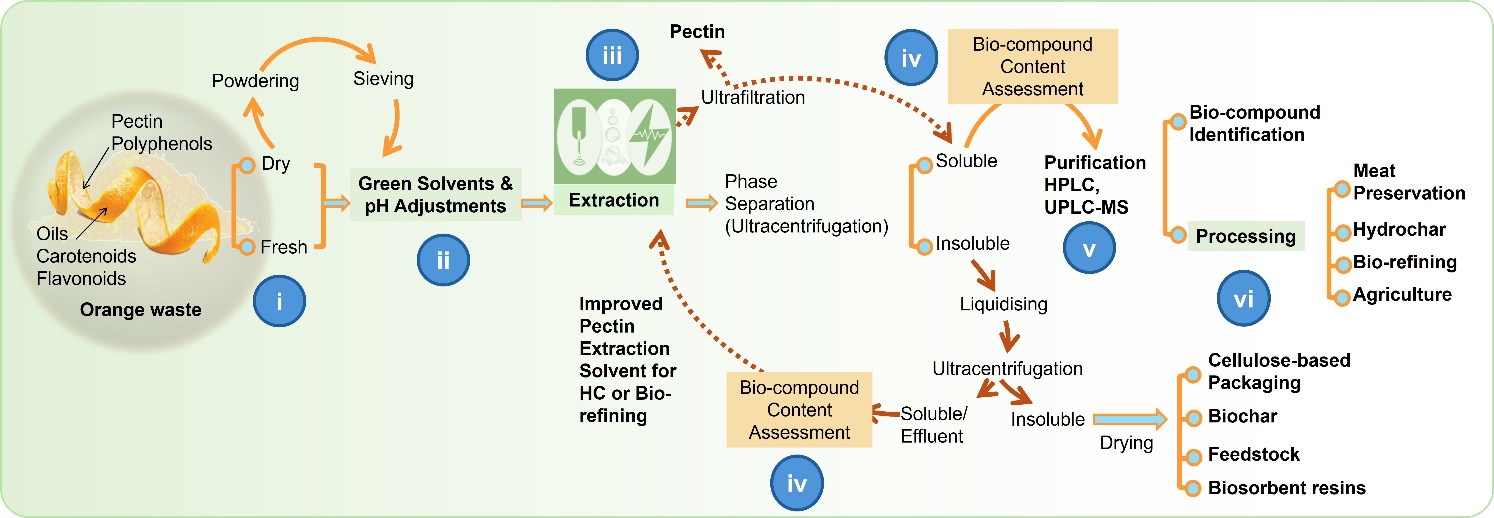


**Supplementary Figure S1**. Typical valorisation scheme involving green technologies. i; peel drying and pulverizing, ii; dried peel mixing with chosen green solvent, iii; technology-assisted extraction, iv; extract content determination, v; bioactive compound identification and purification, and vi; processing and valorisation. HPLC, high performance liquid chromatography; UPLC-MS, ultra-performance liquid chromatography-tandem mass spectrometry.


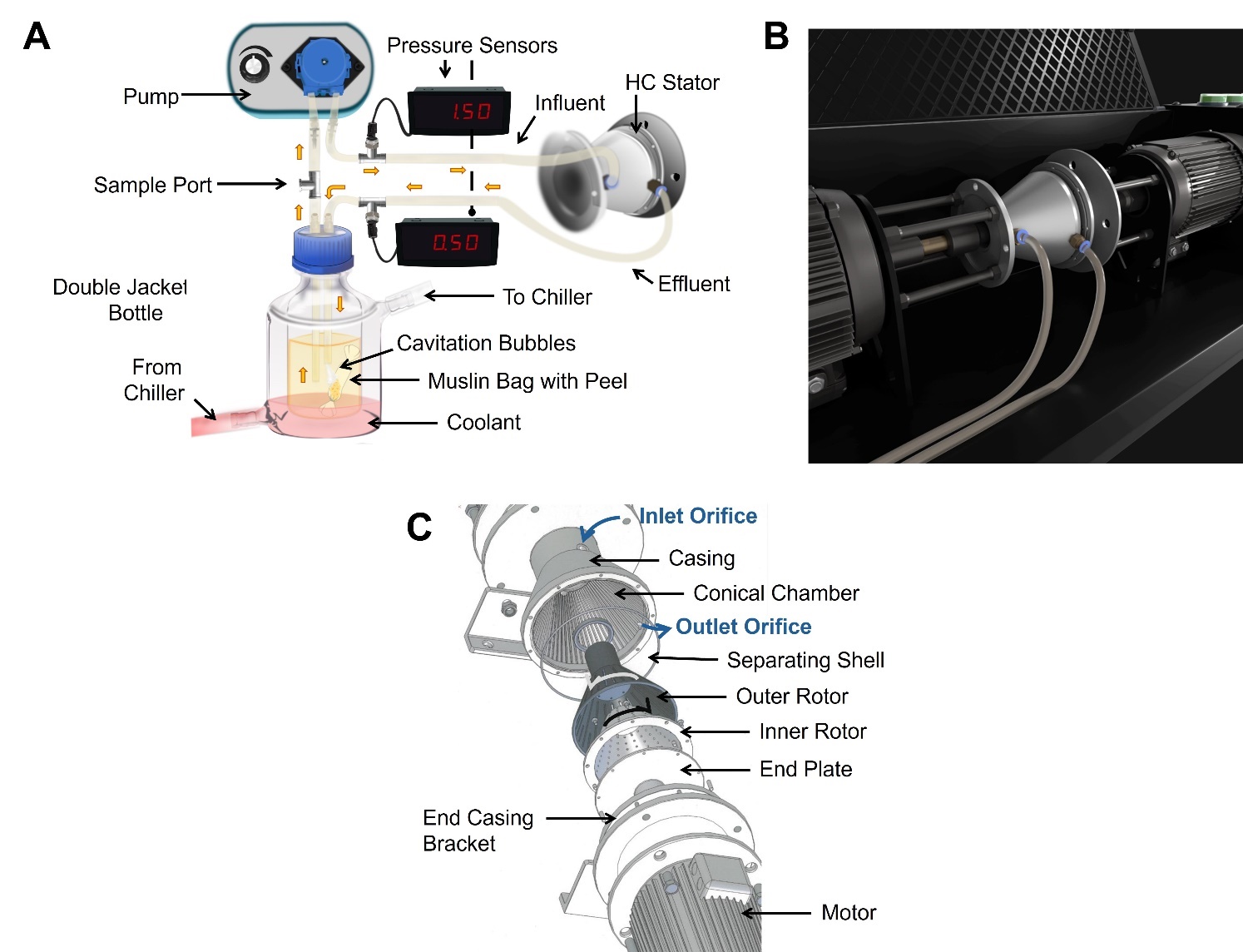


**Supplementary Figure S2**. HC-assisted extraction set-up (A). HC-stator in its casing. Influent and effluent tubings are visible (B). Compartment organization within the HC stator. Interspaces between inner/outer rotors and outer rotor/casing are 3 mm (Isopo, 2010).


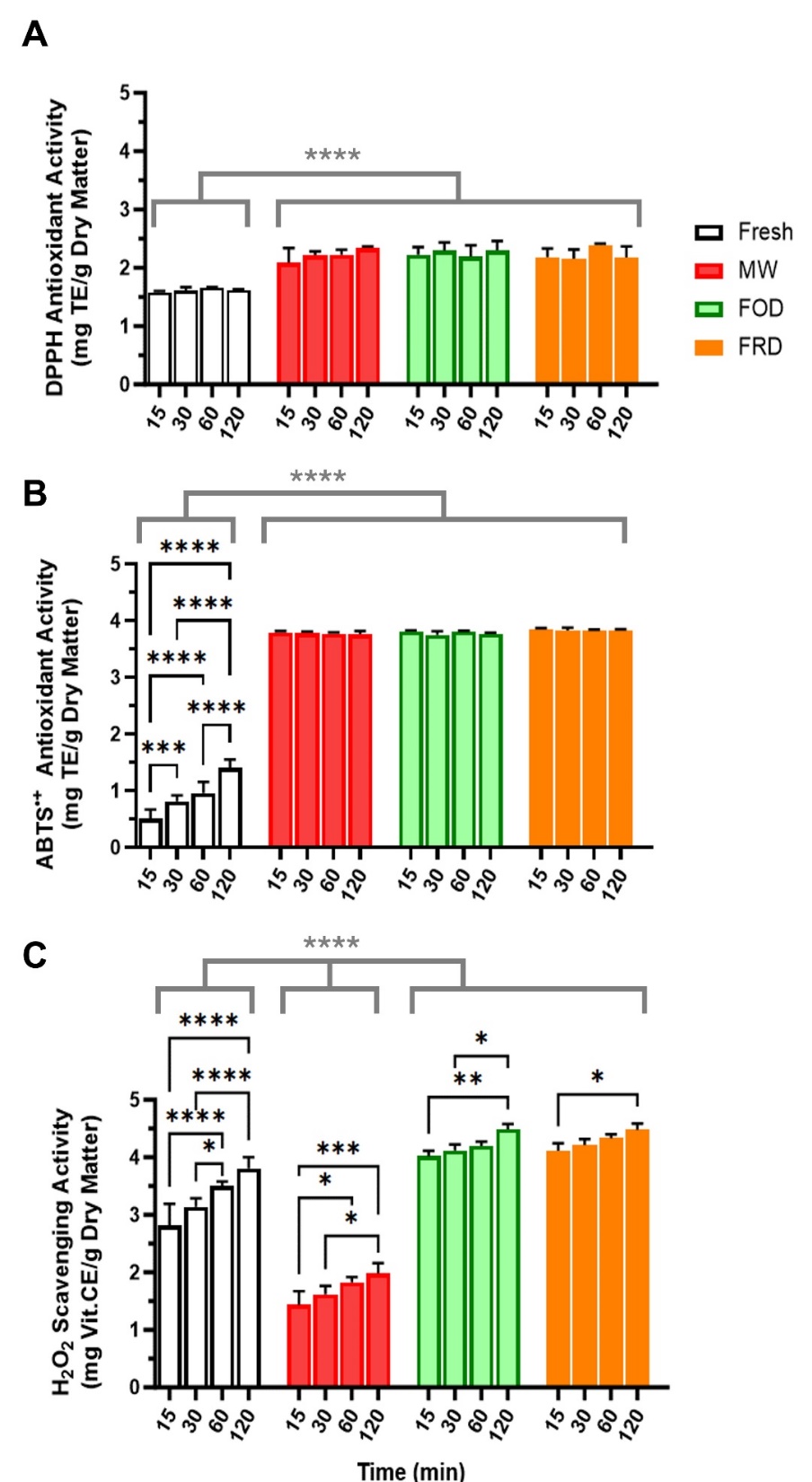


**Supplementary Figure S3**. DPPH (A), ABTS^•+^ (B), and H_2_O_2_ scavenging (C) activities of fresh and dried peel, in 70% (v/v) ethanol (conventional extraction; maceration), at 35^o^C, using 1:40 solid to liquid ratio (g/ml) over time. MW; microwave dried, FOD; hot-air dried, and FRD; freeze-dried. Note that fresh peel weight was normalized to dry weight, considering the moisture percentage loss of the dried peel. Bars denote STDEV of triplicate measurements. *; *P*>0.05, **; *P*=0.0030-0.0055, ***; *P*=0.0001-0.0007, and ****; *P*<0.0001 (E).


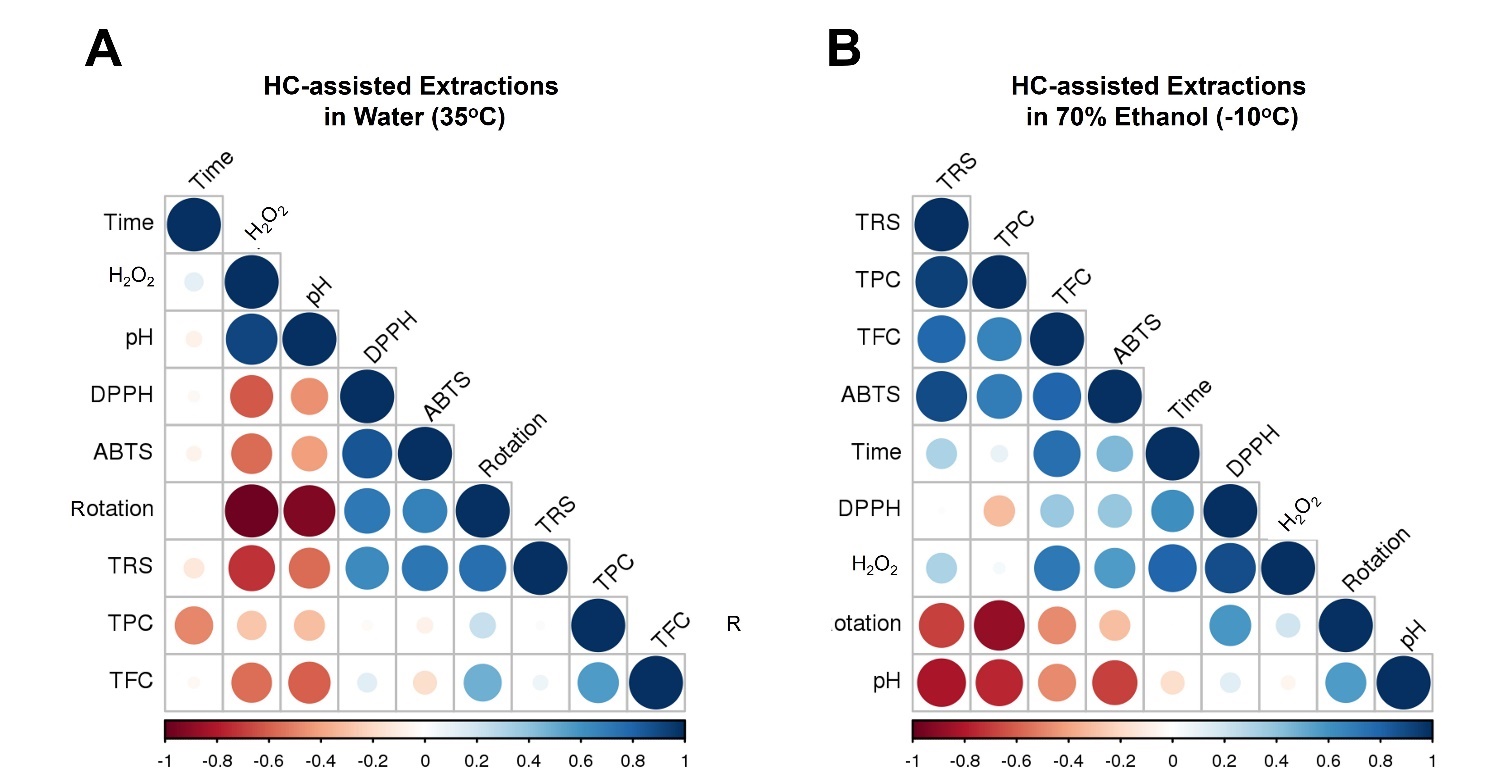


**Supplementary Figure S4**. Pearson correlation matrix on selected parameters. HC in water (A). HC in ethanol (B). H_2_O_2_ refers to the scavenging assay. Graphics were produced using the online STHDA (Kassambara, 2017) R tool.


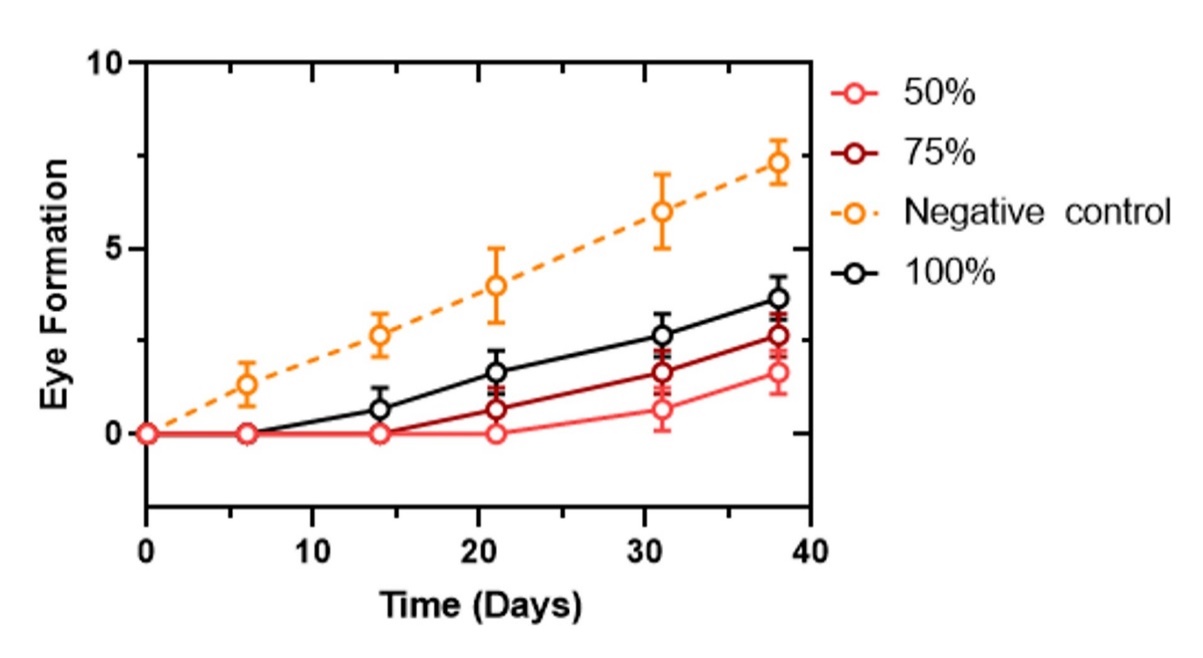


**Supplementary Figure S5.** Eye & sprout formation per spring potato over time. HC-treated peel in water (2 hrs at different rotational frequencies) was washed, dried, and applied on the potatoes (section 2.5). For the negative control, potatoes were treated with distilled water. Error bars denote the STDEV of triplicate measurements (3 spring potatoes).

| **Nitrate Adsorption** | | **Langmuir*** | | | **Freundlich^ǂ^** | | |  |
| --- | --- | --- | --- | --- | --- | --- | --- | --- |
| **State (Orange peel)** | **pH** | **q_max_**  **(mg/g)** | **K_L_**  **(l/mg)** | **R^2^** | **K_f_**  **(mg/g)(L/mg)^1/n^** | **n** | **R^2^** | **Source** |
| Chitosan/Fe/activated carbon. Binding: RT, 60 min | 2.0 | 263.2 | 0.0349 | 0.999 | 8.8157 | 1.16 | 0.977 | Amirsadat et al., 2022 |
| Unmodified (Dried 100^o^C, 24 hrs). Binding; RT, 3 hrs | - | 55.8 | 0.0078 | 0.770 | n.d. | n.d. | n.d. | Shahaji et al., 2023 |
| Acid & Carbon activation. Binding; 30^o^C, 1 hr | 4.0 | 69.4 | 0.0118 | 0.994 | 1.042 | 1.19 | 0.980 | Kumar and Raju, 2020 |
| Unmodified (dried 60^o^C, 24 hrs) | 2.0 | 26.3 | 0.0177 | 0.114 | 0.5728 | 1.19 | 0.829 | Abdulrazak, 2016 |
| Burnt (500^o^C for12 hrs) | 2.0 | 16.12 | 0.0876 | 0.612 | 1.5031 | 1.42 | 0.918 | Abdulrazak, 2016 |
| Maceration (H_2_O), dried 35^o^C, 16 hrs. Binding; RT, 1 hr | 6.0 | 2.13 | 0.0104 | 0.978 | 0.0469 | 1.23 | 0.985 | This work |
| HC-treated (50%, in water, 35^o^C for 2 hrs). Binding; RT, 1 hr | 6.0 | 2.52 | 0.0113 | 0.994 | 0.0477 | 1.33 | 0.985 | This work |
| HC-treated (75% in water, 35^o^C for 2 hrs). Binding; RT, 1 hr | 6.0 | 2.60 | 0.0159 | 0.993 | 0.0616 | 1.62 | 0.991 | This work |
| HC-treated (100% in water, 35^o^C for 2 hrs). Binding; RT, 1 hr | 6.0 | 2.68 | 0.0192 | 0.996 | 0.0681 | 1.33 | 0.982 | This work |
| **Nitrite Adsorption** | | **Langmuir** | | | **Freundlich** | | |  |
| **State (Orange peel)** | **pH** | **q_max_**  **(mg/g)** | **K_L_**  **(l/mg)** | **R^2^** | **K_f_**  **(mg/g)(L/mg)^1/n^** | **n** | **R^2^** | **Source** |
| Maceration (H_2_O), dried 35^o^C, 16 hrs. Binding; RT, 1 hr | 7.0 | 7.177 | 0.0912 | 0.897 | 2.513 | 5.29 | 0.971 | This work |
| HC-treated (50%, in water, 35^o^C for 2 hrs). Binding; RT, 1 hr | 7.0 | 8.041 | 0.1015 | 0.925 | 2.748 | 5.08 | 0.977 | This work |
| HC-treated (75% in water, 35^o^C for 2 hrs). Binding; RT, 1 hr | 7.0 | 9.938 | 0.0901 | 0.924 | 3.587 | 5.78 | 0.933 | This work |
| HC-treated (100% in water, 35^o^C for 2 hrs). Binding; RT, 1 hr | 7.0 | 13.12 | 0.0092 | 0.989 | 0.613 | 2.06 | 0.999 | This work |

**Supplementary Table S1**. Isotherm parameters for NO_3_^-^ & NO_2_^-^ adsorption onto orange peel waste.

^*^Langmuir: q_max_; maximum adsorption capacity, and K_L_; Langmuir constant (free adsorption energy).

^ǂ^ Freundlich: K_f_; adsorption capacity of heterogeneous sites, n; Freundlich exponent for favourability of adsorption and/or heterogeneity of the system (n>1 favours binding).

| **Cu^2+^ adsorption** |  | **Langmuir*** | | | **Freundlich^ǂ^** | | |  |
| --- | --- | --- | --- | --- | --- | --- | --- | --- |
| **State (Orange peel)** | **pH** | **q_max_**  **(mg/g)** | **K_L_**  **(l/mg)** | **R^2^** | **K_f_**  **(mg/g)(L/mg)^1/n^** | **n** | **R^2^** | **Source** |
| Combustion. Binding; 120 min, RT | 6.5 | 16.64 | 0.47 | 0.917 | 5.28 | 2.71 | 0.923 | Safari et al., 2019 |
| Biochar. Binding; RT, 6 hrs | 5.0 | 72.99 | 9.13 | 0.95 | 47.06 | 8.59 | 0.80 | Amin et al., 2019 |
| Saponification. Binding; RT, 2 hrs | 5.0 | 270.3 | 0.075 | 0.971 | 68.11 | 3.45 | 0.835 | Özkan et al., 2017 |
| Unmodified (dried 80^o^C). Binding; 30^o^C, 3 hrs | 5.0 | 44.28 | 0.019 | 0.992 | 3.67 | 2.36 | 0.965 | Feng et al., 2009 |
| Methyl acrylation polymer. Binding; 30^o^C, 3 hrs | 5.0 | 289.0 | 0.033 | 0.993 | 18.93 | 0.50 | 0.826 | Feng et al., 2009 |
| Unmodified (dried 55^o^C). Binding; RT, 24 hrs | 5.0 | 22.10 | SIPS isotherm fits  (Combination of  Langmuir & Freundlich) | | | 0.70 | 0.991 | Izquierdo et al., 2013 |
| Base activated. Binding; RT, 24 hrs | 5.0 | 47.05 |  |  |  | 0.76 | 0.997 | Izquierdo et al., 2013 |
| Base-acid activated. Binding; RT, 24 hrs | 5.0 | 86.73 |  |  |  | 1.26 | 0.992 | Izquierdo et al., 2013 |
| Acid activated (dried 65^o^C). Binding; RT | 6.0 | 4.80 | 0.06 | 0.993 | 4.37 | 3.11 | 0.882 | Surovka and Pertile, 2017 |
| Mercapto-acetic acid treated (dried 70^o^C, 24 hrs). Binding; RT, 30^o^C, 2 hrs | 5.0 | 34.72 | 0.239 | 0.982 | 11.21 | 3.31 | 0.973 | Amin et al., 2017 |
| Unmodified (dried 105^o^C, 24 hrs). Binding; RT, 30-40 min | 5.0 | 38.88 | 0.004 | 0.999 | 2.997 | 2.90 | 0.996 | Romero-Cano et al., 2016 |
| Ethanol washed (dried 105^o^C, 24 hrs). Binding; RT, 30-40 min | 5.0 | 48.95 | 0.006 | 0.997 | 5.142 | 3.22 | 0.998 | Romero-Cano et al., 2016 |
| Pressurised & decompressed (dried 105^o^C, 24 hrs). Binding; RT, 30-40 min | 5.0 | 32.58 | 0.005 | 0.998 | 3.729 | 3.48 | 0.999 | Romero-Cano et al., 2016 |
| Decompressed & acid activated (dried 105^o^C, 24 hrs). Binding; RT, 30-40 min | 5.0 | 163.01 | 0.002 | 0.995 | 4.025 | 0.47 | 0.988 | Romero-Cano et al., 2016 |
| Unmodified. Binding; RT, 1 hr | 5.5 | 14.7 | 0.091 | 0.962 | 2.808 | 2.55 | 0.987 | This work |
| HC-treated (75% in water, 35^o^C for 2 hrs). Binding; RT, 1 hr | 5.5 | 23.8 | 0.059 | 0.970 | 2.702 | 1.96 | 0.932 | This work |

**Supplementary Table S2**. Isotherm parameters for Cu^2+^ adsorption onto orange peel waste.

^*^Langmuir: q_max_; maximum adsorption capacity, and K_L_; Langmuir constant (free adsorption energy).

^ǂ^Freundlich: K_f_; adsorption capacity of heterogeneous sites, n; Freundlich exponent for favourability of adsorption and/or heterogeneity of the system (n>1 favours binding).
